# Clinical and neuroimaging characteristics of diabetic striatopathy: a case series report

**DOI:** 10.3389/fendo.2024.1429026

**Published:** 2024-12-10

**Authors:** Yaning Chen, Chunliu Wu, Ming Ren, Qingjun Wang, Zhiwei Wang, Yimo Zhang, Yingxin Yu

**Affiliations:** ^1^ Department of Endocrinology, Sixth Medical Center of Chinese People's Liberation Army (PLA) General Hospital, Beijing, China; ^2^ Department of Neurology, First Medical Center of Chinese People's Liberation Army (PLA) General Hospital, Beijing, China; ^3^ Department of Neurology, Shanghai Blue Cross Brain Hospital, Shanghai, China; ^4^ Department of Radiology, Sixth Medical Center of Chinese People's Liberation Army (PLA) General Hospital, Beijing, China

**Keywords:** diabetic striatopathy, hemichorea, artery stenosis, hyperglycemia, ketonuria

## Abstract

**Background:**

Diabetic striatopathy (DS) is a rare disorder characterized by clinical manifestations of hemichorea, non-ketotic hyperglycemia, and high signal on T1-weighted MRI or high density on CT scan in basal ganglia, typically associated with poor glycemic control.

**Objective:**

This study aimed to analyze clinical characteristics of patients with diabetic striatopathy to raise awareness amongst physicians, especially endocrinologists, about this rare neurological manifestation in patients with diabetes.

**Methods:**

We retrospectively analyzed the data on clinical presentations, laboratory workups, and cranial CT and MRI of six patients with DS who were admitted to our hospital from October 2013 to June 2022.

**Results:**

The mean age of onset among the six patients was 80.5 years, and the mean value of HbA1c was 13.65%. All six patients complained of involuntary movements, which primarily affected the arm and leg on one side of the body. Bilateral caudate nucleus hyperdensities were shown on the CT examination in Case 3,while the other 5 patients, unilateral caudate nucleus hyperdensity was shown. In addition, five patients (except Case 5) underwent MRI, all showing hypersignal lesions on the T1-weighted images. A low signal in the right basal ganglia was shown on MRI susceptibility weighted imaging (SWI) sequences in Case 6. All six patients exhibited carotid artery or cerebral artery stenosis. Following strict blood glucose control and symptomatic management, the symptoms of chorea improved significantly in all patients, and repeat images indicated that the lesions gradually disappeared.

**Conclusion:**

Both poor vascular conditions and severe hyperglycemia contribute to the development of diabetic striatopathy. The prognosis is usually good by active treatment.

## Introduction

The term diabetic striatopathy (DS), a central nervous system disorder limited to striatal microangiopathy, was proposed in 2009 (1) and is also known as “hemichorea associated with non-ketotic hyperglycemia” or “hyperglycemic non-ketotic hemichorea” and was reported for the first time in 1960 (2). DS is a rare chronic complication of diabetes mellitus with an incidence of 1 in 100,000 (3). It is characterized by non-ketotic hyperglycemia, striatal hyperdensity on computed tomography (CT) or hyperintensity on T1-weighted magnetic resonance imaging (MRI) and sudden onset of hemichorea (4). Hemichorea consists of involuntary, rhythmic, aimless, jerky movements involving distal limbs. This condition affects the quality of daily life for patients and may also lead to death. Due to its rarity and similarities to other conditions, such as intracranial hemorrhage, DS is often misdiagnosed or overlooked. Early recognition and treatment can improve prognosis. We designed this study to draw the attention of more clinicians’ to this disease and to avoid misdiagnosis or underdiagnosis. In this study, we describe six patients with DS and analyze their clinical characteristics to raise awareness among physicians about this rare neurological manifestation.

## Materials and methods

### Patients

We retrospectively analyzed the clinical and laboratory data of six patients with DS admitted to our hospital from October 2013 to June 2022. The Ethics Committee of the Sixth Medical Center of Chinese PLA General Hospital approved this study(No: HZKY-PJ-2024-15). The CT and MRI images were examined by two radiologists who were unaware of the patients’ clinical data. Diagnosis was based on symptoms, laboratory tests, and imaging.

### Definition

In the present study, diabetic striatopathy (DS) was defined as a hyperglycemic condition associated with either one or both of the following conditions: (1) chorea/ballism; (2) hyperdensity observed on CT or hyperintensity on T1-weighted MRI as previously reported (5).

## Results

### Clinical features

Six patients, three females and three males, were diagnosed with DS in our hospital from October 2013 to June 2022. None of them had a family history of movement disorders, exposure to potentially harmful drugs, Wilson’s disease, or polycythemia. Clinical profiles and examination results are summarized in **Table 1**.

**Table 1:** Clinical characteristics of all cases.

Case No.	Sex	Age (y)	Type of DM	Pre-treatment interval	Side of chorea	EEG	HBP	CI	MI	CH	CAS/VAS	HbA1c%	HCY	TC	TG	LDL	HDL	Cr	UAB	Therapy	Outcome
1	F	86	2	5m	Left		–	+	–	+	+	10.5	22.9	5.2	1.66	2.91	1.39	133.4	–	Haloperi dol	disappear
2	F	76	2	9d	Right	N	+	+	–	–		11.8	15	5.34	2.74	3.77	0.77	59.5	–	Haloperi dol	disappear
3	M	66	2	36d	Right	N	+	+	–	+	+	17.3	12.2	2.46	0.51	1.26	0.87	67.5	–	Haloperi dol	improve
4	F	88	2	15d	Left	I	+	+	+	–	+	15	11.5	5.28	1.81	3.61	1.13	72.3	+	No	disappear
5	M	83	2	20d	Left		+	–	–	–	+	12.5	12.2	4.76	1.03	3.1	1.25	71.9	+	tiapride	improve
6	M	84	2	5d	Left	I	+	+	+	+	–	14.8	23.3	3.35	0.78	1.38	1.56	152.6	–	No	disappear

F, female; M, male; EEG, electroencephalogram (“N” means “normal”. “I” means “abnormal”.); HBP, hypertension; CI, cerebral infarction; MI, myocardial infarction; CH, cerebral hemorrhage; CAS/VAS, carotid artery stenosis and/or vertebral artery stenosis; HCY(umol/L), homocysteine; TC(mmol/L), total cholesterol; TG(mmol/L), triglyceride; LDL(mmol/L), low density lipoprotein; HDL(mmol/L), high density lipoprotein; Cr(umol/L), creatinine; UAB, urine acetone bodies.

The mean age of onset was 80.5 years, ranging from 66 years to 88 years. The pre-treatment course of hemichorea ranged from 5 days to 5 months. All six patients admitted initially complained of involuntary movements. Same-side arm-leg combination movement, as previously described, was observed in four of the cases. Case 2 exhibited an arm-leg-face combination while Case 5 exhibited only an isolated leg in. The ratio of left to right involvement was 4:2. No bilateral involvement was noted.

All six patients were diagnosed with type 2 diabetes mellitus. In Case 5, involuntary movement was the first symptom of diabetes mellitus. The average value of glycated hemoglobin A1c(HbA1c) in the six patients was 13.65%, ranging from 10.5% to 17.3%. All of the patients were diagnosed with non-ketotic hyperglycemia, except two patients found with ketonuria. The levels of serum creatinine in Case 1 and Case 5 were slightly higher than normal. All of them had a normal liver function. None of the patients had a lumbar puncture. The electroencephalogram (EEG) of Case 2 and Case 3 showed normal activity. Alpha and beta waves were observed in awake and calm states in Case 4 and Case 6.

### Vascular conditions

**Figure 1:**
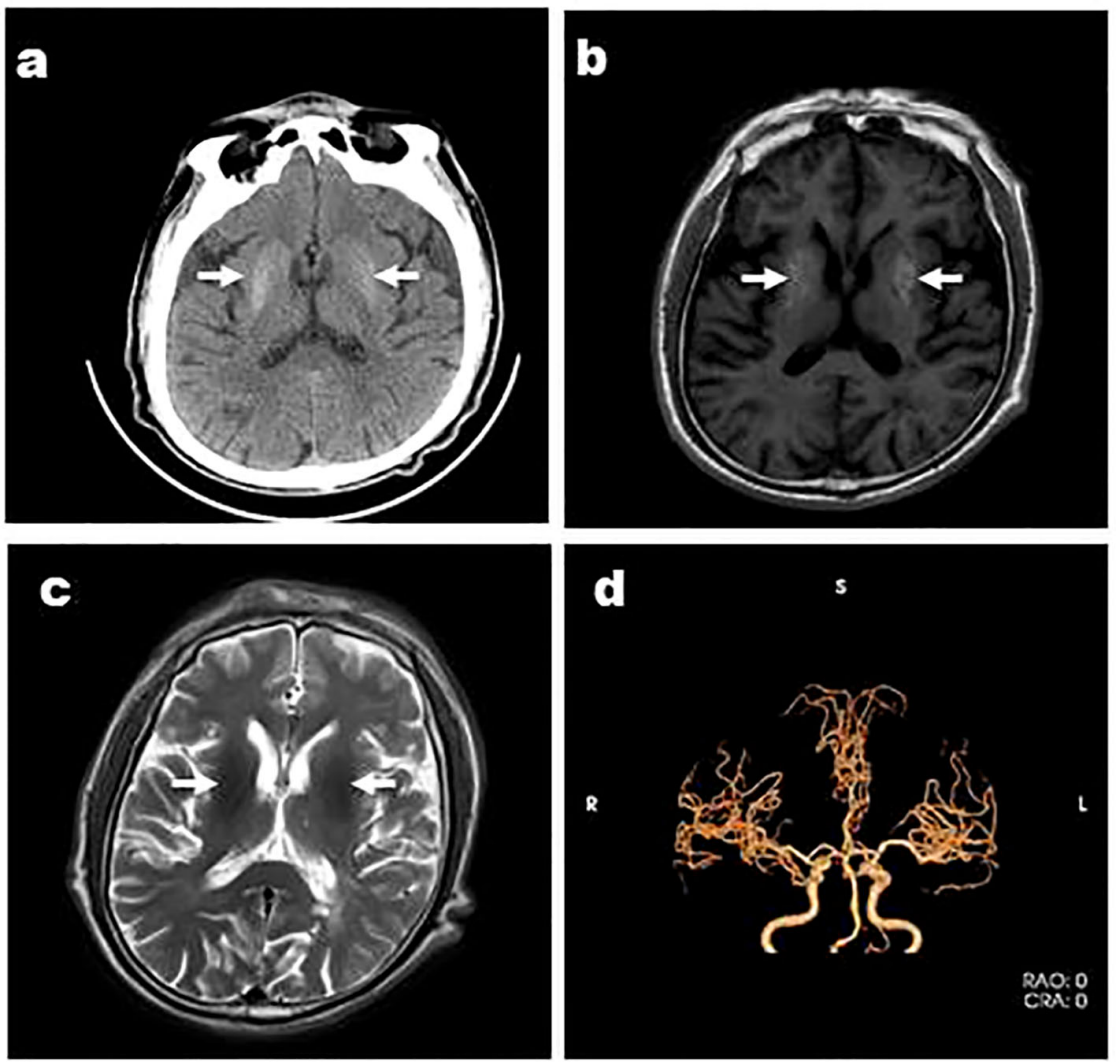
Case 3, a 66-year-old male, was admitted with “involuntary movement of the tongue and right limb for more than 1 month” and images showed bilateral striatum high density on CT **(A)**, high signal on T1WI **(B)**, iso-signal on T2WI **(C)**, and MRA showed arterial stenosis **(D)**. Arrows indicate bilateral striatum abnormal signal.

**Figure 2:**
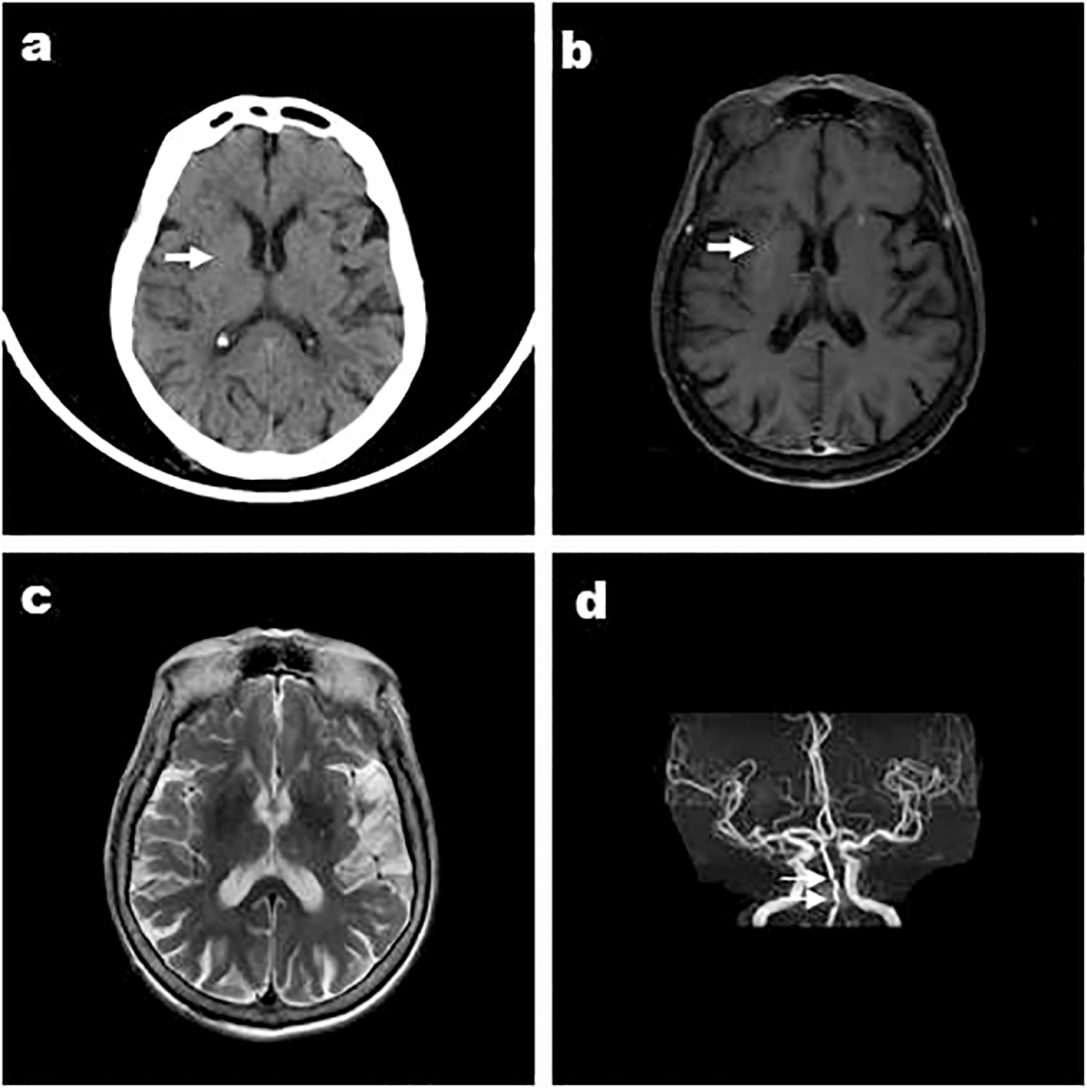
Case 4, an 88-year-old female, was admitted to hospital with “involuntary movements of the left limb for 15 days” and images showed that the right striatum of involuntary movements was high density on CT **(A)**, high signal on T1WI **(B)** Arrows indicate arterial stenosis, iso-signal on T2WI **(C)**, and MRA showed arterial stenosis **(D)**. Arrows indicate bilateral striatum abnormal signal.

**Figure 3:**
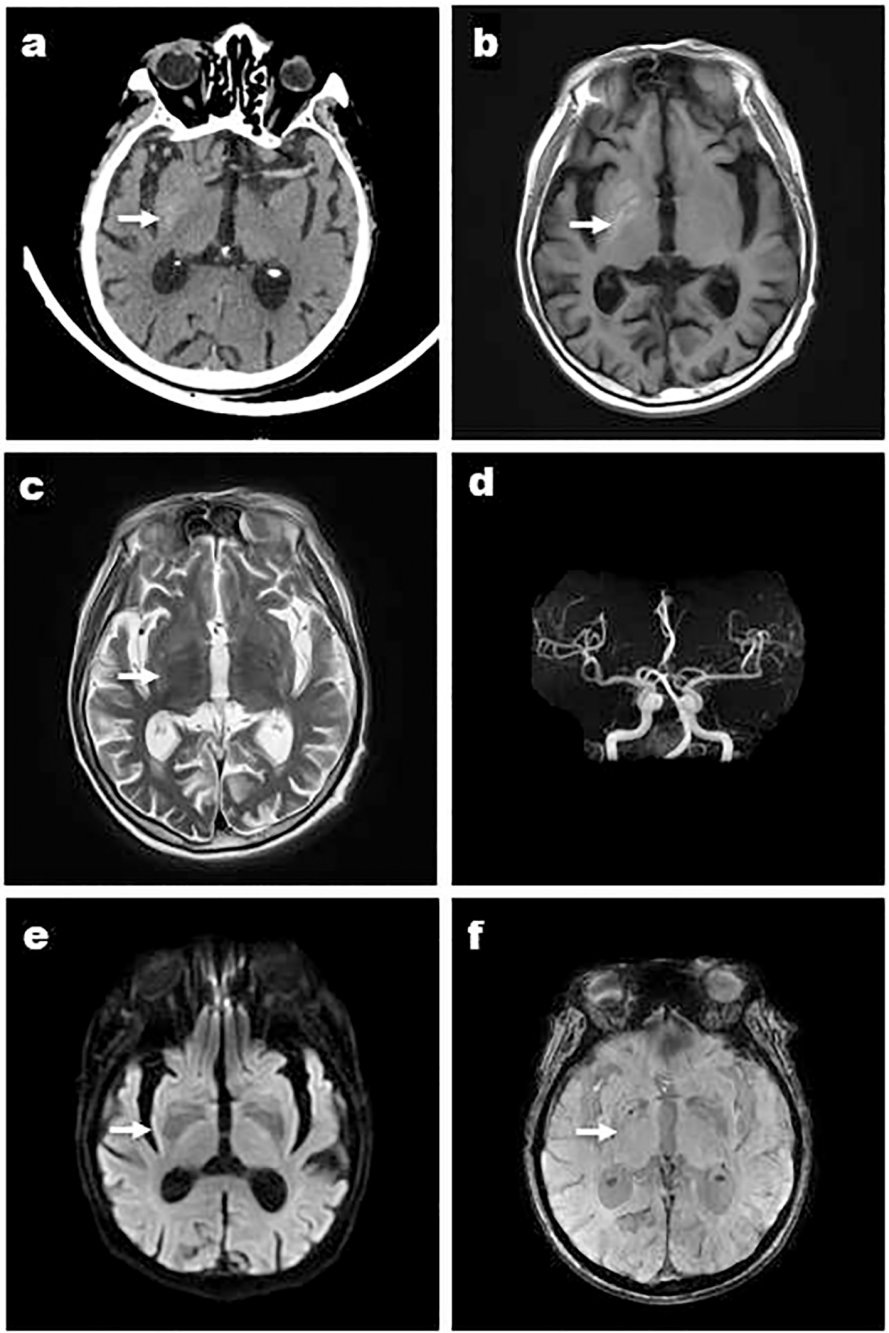
Case 6, a 84-year-old male, was admitted with “left limb involuntarily writhing for 5 days”and images showed that the right striatum of involuntary movements was high density on CT **(A)**, high signal on T1WI **(B)**, iso-signal on T2WI **(C)**, and MRA showed arterial stenosis **(D)**. DWI didn’t show high signal **(E)** and a low signal in the right basal ganglia was shown in susceptibility weighted imaging (SWI) **(F)**. Arrows indicate striatum abnormal signal.

Five of six patients had hypertension. Carotid artery stenosis and/or vertebral artery stenosis appeared in Cases 1, 3, 4, and 5. Anterior, middle, and/or posterior cerebral artery stenosis was present in Case 2, 3 (**Figure 1D**), 4 (**Figure 2D**), 5, and 6(**Figure 3D**). A previous history of cerebral infarction was noted in Cases 1, 3, 4, and 6while Case 2 had a concurrent new cerebral infarction. Imaging examinations showed old bleeding in the corresponding areas in Cases 1, 3 and 6. Myocardial infarction had previously occurred in Cases 4 and 6.

### Imaging features

Except Case 5, all the cases underwent cranial CT and MRI examinations (**Figures 2**, **3**). The characteristic images, hyperdensity on CT (**Figures 1A**, **2A**, **3A**) and high signal intensity on MRI T1-weighted sequences (**Figures 1B**, **2B**, **3B**) in the striatum, contralateral to the involuntary movement, were shown in Cases 1,2, 4, and 6. The signals on MRI T2-weighted sequences were variable (**Figures 1C**, **2C**, **3C**). In Case 3, hyperdensity in CT and hyperintensity in the T1-weighted image were shown on the bilateral striatum. In Case 5,the patient only had a cranial CT scan that showed a slight hyperdensity in the right basal ganglia. Normal signal in the right basal ganglia was shown in MRI diffusion weighted imaging (DWI) sequences in Case 6 (**Figure 3E**). A low signal in the right basal ganglia was shown in MRI susceptibility weighted imaging (SWI) sequences in Case 6 (**Figure 3F**).

### Prognosis

All patients received hypoglycemic therapy. At the same time, oral haloperidol was prescribed for Cases 1, 2, and 3 and tiapride for Case 5. Hemichorea disappeared when Cases 1, 2, 4, and 6 were discharged after treatment. The conditions of case 3 and 5 improved when they left the hospital.

## Discussion

In our study, six patients were enrolled because of hemichorea associated with hyperglycemia and were diagnosed with DS by clinical features, symptoms, and imaging. Involuntary movements resolved after glucose control and/or combination with dopamine-inhibiting drugs.

Six patients were finally diagnosed with DS after other causes of the disease were excluded, such as cerebrovascular disease, Huntington’s disease, hepatolenticular degeneration, thyroid disease, parathyroid disease, infectious disease, chronic liver disease, and poisoning. Among all six patients with a mean onset age of 80.5 years(range, 66–88), four cases(66.7%) were in their eighth decade of life. In the study by Chua CB, et al., 176 patients (male:female=1:1.7) were identified from 72 articles with a mean age of 67.6 ± 15.9 (ranging 8–92) (3). The age range of our patients is similar to previous studies, but more than half of the patients were over 80 years old. The average age is affected by many factors, such as the included population and the number of patients. The age distribution characteristics of this disease should be of greater concern and should be analyzed further.

DS occurs predominantly in patients with long-standing poorly controlled type 2 diabetes mellitus (3), rarely in type 1 diabetes mellitus and even more rarely in type 3C diabetes (6). They had poor glycemic control, with remarkable elevation of blood glucose and HbA1c. It was reported that 17% of 176 patients with DS were newly diagnosed with diabetes (3). In our study, DS was the first presentation of diabetes mellitus in Case 5. Therefore, we recommend that a blood glucose test should be performed in those patients with hemichorea. DS was evenly distributed between genders, which is consistent with the study by Cosentino C et al. (7). In terms of the body regions affected, arm-leg extremity involvement appeared with the highest frequency (3). Similar results were obtained in our study.

One of the typical clinical manifestations of DS is non-ketotic hyperglycemia. However, in our study, urinary ketone bodies were positive in Case 4 and 5. Mikulenka P and Stetkarova I (8) also reported an instance of a 90-year-old male that was diagnosed with hemichorea and ketotic hyperglycemia with a hyperdense striatum. Safan AS et al. (9) also reported a patient who presented with focal seizures in the context of ketotic hyperglycemia. In a study of 71 patients who were tested for ketone bodies, 18.3% were positive for ketonemia or ketonuria (10). Therefore, DS can also occur in patients with ketotic hyperglycemia.

CT and MRI are the two most commonly used imaging methods to detect striatal anomalies of DS. There is a very clear correlation between their imaging features. Striatal hyperdensities on brain CT and/or hyperintensities on brain T1-weighted MRI appear contralateral to hemichorea in patients with unilateral syndrome. However, in our study, Case 3 presented with unilateral chorea and bilateral striatal imagining abnormalities, which had been previously reported (7). Typical neuroimaging abnormalities without typical symptoms of DS movement disorders have also been reported in 2% of patients (11). In addition, Chua et al. found that nearly 7% of patients with chorea did not show striatal involvement on neuroimaging (3). Therefore, Dubey et al. proposed to categorize DS into three subsets: symptomatic DS, clinically isolated DS, and radiologically isolated DS (11). In our study, all six cases can be categorized into the symptomatic DS subgroup, and Case 3 may also include radiologically isolated DS due to abnormalities in the bilateral striatal imagery and unilateral chorea.

Currently the pathogenesis of DS is unclear and recently Chatterjee et al. proposed a theory of pathophysiology of DS(a dozen of demons), but namely ischemia, blood-brain barrier dysfunction and neuroinflammation, oxidative stress and neurotoxicity, gemistocytopathy and astrocytic dysfunction, microhemorrhages and methemoglobin deposition, paramagnetic mineral deposition, cytotoxic edema and cellular swelling, myelinolysis, autoimmune and infectious etiology, gliosis, atrophy and structural changes and metabolic theory. These factors are closely interrelated and overlapping (12).

Therefore, this study focuses on a few of these pathophysiologic theories.

(1) **Metabolic disorder theory.** When hyperglycemia appears, the brain cells shift toward the anaerobic pathway of glucose metabolism, choosing gamma-aminobutyric acid (GABA) and acetate as energy sources, possibly resulting in metabolic acidosis, and further contributing to basal ganglia dysfunction. However, hyperglycemia is a systemic disease that affects bilateral metabolic disorders but the theory of systematic metabolic disorders is insufficient when trying to explain the unilateral-onset chorea. In addition, chorea can also occur after hypoglycemia or correction of blood glucose. Therefore, in a sense, hyperglycemia is not the only potential cause of DS (13).

(2) **Theory of bleeding injury.** At present, microhemorrhage theory has also been proposed to elucidate the pathogenesis of DS. However, unlike conventional cerebral hemorrhage, DS lesions are confined to the striatum, and generally do not involve the thalamus or the internal capsule and there are no edema or occupancy effects. In addition, the high signal duration of T_1_WI on magnetic resonance is longer and the signal evolution of T_2_WI is not consistent with the evolution characteristics of conventional hematoma (14). It is suggested that the mechanism of the abnormal signal is different from that of conventional cerebral hemorrhage.

(3) **Ischemic injury theory**. γ -Aminobutyric acid (GABA)-ergic medium-sized spiny neurons (MSN), chronic hyperglycemia, and micro- and macroangiopathy may lead to hypoperfusion and subsequent ischemia within the basal ganglia (12). Ischemic theory has been supported by some biopsies. Ischemia causes low oxygen and a decrease in local cerebral blood flow state, which inhibits the Krebs cycle, leading to a significant decrease in GABA levels and hence, a relatively higher dopaminergic activity, which causes a disinhibition of the subthalamic nucleus and thus an excessive activation of the motor cortex by thalamic projections (12, 17). Previous studies of biopsies of the patients’ basal ganglia brain tissues showed thickening and occlusion of each layer of arterioles, accompanied by patchy necrosis, neovascularization and paravascular inflammation, suggesting that ischemia in the striatum region was related to the development of lesions (1). In addition to some autopsy findings, specific imaging studies also support striatal ischemia or infarction in DS patients. Some authors reported decreased perfusion in the involved basal ganglia in DS patients by single photon emission computed tomography (SPECT) (15). Magnetic resonance spectroscopy can also help monitor striatal ischemic injury in DS patients if elevated lactate and N-acetylaspartate depletion is observed (13). Further evidence from SWI (susceptibility-weighted MR imaging) supported ischemic infarction with reactive gemistocyte proliferation and mineral deposition as the etiology of hemichorea, but not petechial hemorrhages (16). In our study, six patients with poor intracranial vascular conditions were at very high risk of ischemic stroke in view of age, diabetes, hypertension, carotid and/or vertebral artery stenosis, anterior, middle and/or posterior cerebral artery stenosis, and cerebral infarction.

The metabolic and vascular theories are very important theories that hyperglycemia causes DS (17). All six patients in this study suffered from hyperglycemia and vascular disease and were at high risk for cerebral infarction. We speculate that striatal vascular stenosis underlies the pathology of DS, with hyperglycemia as the pathological driver.

A quarter of patients achieved the resolution of clinical symptoms by glycemic control alone (3). In our study, hemichorea disappeared in Cases 4 and 6 only by glycemic control. However, recurrence of same-side hemichorea on of those previously affected has been reported in some patients after stopping anti-chorea drugs over two months and two years after the first episode of chorea (10). Even after the neuroimaging abnormalities have been resolved, the recurrence rate is relatively high at about 20% (3). Therefore, regular follow-up is necessary regardless of neuroimaging results.

Our study has several limitations. First, the number of cases was relatively small. Second, we did not regularly screen for chronic complications of diabetes. Third, the follow-up period was not long enough.

In conclusion, DS may occur in patients with ketotic hyperglycemia. Typical movement disorders do not always coincide with typical imaging. We speculate that poor vascular conditions and marked hyperglycemia combine to promote the development of DS. It is recommended to test blood glucose levels at the time of diagnosis with hemichorea.

## Data Availability

The raw data supporting the conclusions of this article will be made available by the authors, without undue reservation.
